# Time-Dependent Changes in Performance, Biochemistry, and Histology in Dairy Calves with Acute Aflatoxicosis

**DOI:** 10.3390/vetsci12030273

**Published:** 2025-03-14

**Authors:** María Carolina de Luna-López, Arturo G. Valdivia-Flores, Erika Janet Rangel-Muñoz, Emmanuel Hernández-Valdivia, Teódulo Quezada-Tristán, Fernando Jaramillo-Juárez, Raúl Ortiz-Martínez

**Affiliations:** 1 Agricultural Sciences Centre, Universidad Autónoma de Aguascalientes, Av. Universidad 940, Aguascalientes 20100, Mexico; carolina.deluna@edu.uaa.mx (M.C.d.L.-L.); janet.rangel@edu.uaa.mx (E.J.R.-M.); emmanuel.hernandez@edu.uaa.mx (E.H.-V.); teodulo.quezada@edu.uaa.mx (T.Q.-T.); raul.ortiz@edu.uaa.mx (R.O.-M.); 2 Basic Sciences Centre, Universidad Autónoma de Aguascalientes, Av. Universidad 940, Aguascalientes 20100, Mexico; jara@att.net.mx

**Keywords:** aflatoxins, dairy cows, hepatotoxicity, hematology, histopathology, serum chemistry

## Abstract

Aflatoxins (AFs) are secondary metabolites of *Aspergillus* spp. They are highly toxic, carcinogenic, and immunosuppressive; AFs cause nonspecific disorders in humans and animals, which makes their diagnosis complex. The objective was to describe the time course of toxic effects of a single exposure to AFs-contaminated feed. Fifteen male calves (2 weeks old) were examined over 30 days for clinical, biochemical, and pathological changes resulting from the ingestion of AF-contaminated feed (1.0 mg/kg BW). Compared with 15 unexposed calves, exposed calves showed transient depression and rough coat; BW gain, dry matter intake, albumin, total plasma protein, and hepatic and renal glutathione-S-transferase concentrations progressively decreased. However, conversion ratio (feed/BW), total bilirubin, direct bilirubin, alkaline phosphatase, reduced glutathione, gamma-glutamyltransferase, and alanine and aspartate aminotransferases progressively increased. Necropsy and histology at 7 days postexposure (dpe) showed liver with multifocal hemorrhages, yellowish coloration, friable consistency, periportal fibrosis, and steatosis. Kidneys were hemorrhagic, with brush border losses, glomerular atrophy, sclerotic glomerulonephritis, and lymphocytic infiltration. However, at 30 dpe, the liver showed pale discoloration, diffuse macrovesicular steatosis, and periportal fibrosis. The kidneys had mottled appearance and firm consistency, fibrosis, loss of normal architecture, and thickening of Bowman’s capsule. These results suggest that the identification of alterations in animal performance and biochemical and histological characteristics could be useful for integrating a proper diagnosis of bovine aflatoxicosis.

## Simple Summary

This study describes the time course of the toxic effects of a single exposure to feed contaminated with aflatoxins on performance, biochemistry, and macroscopic and microscopic lesions in Holstein calves raised for meat. These findings are useful for integrating a proper diagnosis of aflatoxicosis in cattle. Diagnosing of aflatoxicosis is highly relevant to the dairy industry to reduce the negative impact of this mycotoxin on animal health and the persistence of its hydroxylated metabolites in raw and pasteurized milk. Therefore, this study serves as a basis for a proper analysis of the occurrence of bovine aflatoxicosis and consequently provides further evidence to support prevention measures to reduce the risk of the human population being exposed to ingestion of aflatoxin metabolites.

## 1. Introduction

Aflatoxins (AFs) are compounds produced by the filamentous fungi *Aspergillus* spp., mainly *A. flavus* and *A. parasiticus*, which exert a wide range of effects on other living organisms [1,2] AFs are the most potent natural toxicants, and they are carcinogenic, mutagenic, and teratogenic compounds. Additionally, they may have immunosuppressive, hepatotoxic, and nephrotoxic effects [3]. When AFs are ingested in contaminated feed, they are rapidly absorbed in the gastrointestinal tract through a passive mechanism and reach a plasma maximum concentration within 35.0 min; the elimination process practically concludes after two days, and AFs are also rapidly removed from tissues a few hours after a single exposure [4]. Absorbed AFs are metabolized by the hepatic system of mixed-function oxidases, which convert them into reactive epoxides (AFBOs). Reactive AFBOs bind to subcellular structures, nucleic acids, and proteins; this adversely affects cellular functions such as protein synthesis, blood clotting, and fat metabolism in the liver. Reactive epoxides also cause subcellular damage and induce the release of membrane enzymes into the bloodstream [5]. The epoxide can be conjugated with reduced glutathione (GSH) through the action of the glutathione-S-transferase (GST) enzymes and form the compound, N-acetylcysteine-AFs, which is eliminated via the biliary and urinary tract; AFs in feed can also be transferred to milk and urine 12 h after feeding as hydroxylated aflatoxin (AFM1 and AFM2) in the 1.0–6.0% range [6,7,8].

The severity of aflatoxicosis is influenced by several factors, including the intake dose and time of exposure to AFs, as well as the animal’s age and breed type and the integrity of the animal’s digestive functions [9]. Cattle are susceptible to AFs intoxication, and dairy cows are the most affected because of their higher metabolic demand associated with milk production and metabolic disorders [10]. In addition, chronic AF intoxication is the most frequent form in mature ruminants because these animals have a presystemic elimination system, carried out by the metabolic activity of the rumen microbiota [11]. Acute aflatoxicosis syndrome occurs in cattle when exposed to high amounts of AFs (1.1–33.5 mg/kg) [12,13], while chronic aflatoxicosis is associated with moderate and continuous ingestion of this mycotoxin [14]. The clinical manifestations of both acute and chronic aflatoxicosis in cattle are not characteristic, but a reduction in productive performance and increased susceptibility to infection are notable. For these reasons, it has been proposed that the diagnosis of aflatoxicosis requires the use of biochemical and histopathological studies [14]. As in many regions of the world, the presence of toxigenic *Aspergillus flavus* in dairy cattle feed is frequent in central Mexico. When feed is formulated and maintains an adequate relative humidity content (8–10%), the growth of the vegetative forms of the fungal microflora is inhibited, although the spores and mycotoxins produced remain stable within the processed material. However, if the feed is hydrated under inadequate storage conditions, AF concentrations can dangerously increase in feed and milk intended for human consumption, causing an increased health risk to human and animal populations [7,14,15]. The objective of this study was to describe the toxic effects of a single exposure to a feed contaminated with aflatoxins on the performance, serum and tissue biochemistry, and macroscopic and microscopic lesions in dairy calves. Thus, it was hypothesized in this study that if calves are exposed to a single dose of aflatoxin, a sequence of time-dependent changes in performance, biochemistry, and macro- and microscopic morphology can be observed, which will be useful for the proper diagnosis of acute aflatoxicosis.

## 2. Materials and Methods

### 2.1. Study Design and Herd Management

The Ethics Committee for the Use of Animals in Teaching and Research of the Autonomous University of Aguascalientes reviewed, approved, and supervised the study to ensure adherence to the approved protocol. This Committee considered it justified to approve the protocol because of the high frequency of AF contamination in the region, both in the feed of dairy cows and in milk, posing significant public and animal health risks. Thirty male Holstein calves were raised for their meat on a large cattle farm in Aguascalientes, central Mexico, following standard calf management practices [16]. Calves were housed during the spring season in individual metal pens (1.8 × 2.4 m) in a rearing room at ambient temperature of 20–26 °C and relative humidity of 54–69%. Calves were fed 2.0 L of milk twice daily using bottles, ensuring a total daily consumption of 4 L of milk per animal and ad libitum access to water and commercial concentrated starter feed. The concentrate starter feed was purchased from the institutional feed mill (18.5% crude protein, 3.7 Mcal/kg of DM of digestible energy, 2.5% raw fat, 15.5% fiber, 3.3% ash, 0.7% Ca, 0.45% P, and 10.0% maximum humidity). At 14 days of age, calves were weighed, sorted according to body weight (50.2 ± 2.0 kg), numbered consecutively from 1 to 30, and randomly assigned into two experimental groups: nones, nonexposed group (NE-G, *n* = 15) and pairs, exposed group (E-G, *n* = 15). The NE-G was fed milk and AF-uncontaminated basal diet (detection limit < 2.0 ng/g), while the E-G received the same feed as the NE-G, plus a single dose of milk with added milled corn contaminated with AFs (1.0 mg/kg BW). All animals were kept under close surveillance until 30 days postexposure (dpe). The BW was recorded at 1, 7, 15, and 30 dpe, and BW gain (BWG) and growth rate (GR) were calculated.

### 2.2. Aflatoxins Production and Quantification

Two Mexican toxigenic isolates of *Aspergillus flavus* were cultured on potato dextrose agar as previously described [1]. The spore suspension was obtained (5 × 10^6^ spores/mL) with Tween 20 solution (0.1%), sterile distilled water, and paraffinic oil (0.1%) as fixative. Corn kernels were inoculated with the spore suspension (2.5 × 10^5^ spores/g grain) following a noninvasive, sterile method. Humidity was adjusted to 15.0% by addition of distilled water. The kernels were incubated at 29.0 ± 2.0 °C for 14 d. The entire procedure was carried out under sterile conditions and in adherence to strict personal safety protocols.

Concentrated feed and unreconstituted milk powder samples were obtained in triplicate directly from the contaminated feed and milk batches. The concentration of AFs was estimated by the 990.33 official method of the Association of Official Analytical Chemists [17] via a fluorescence HPLC system (FP-2020, Varian Associates Inc., Victoria, Australia; LC–18 column, Supelcosil, Supelco Inc. Bellefonte, PA, USA), and solid-phase extraction tubes (SPE; Supelco, Bellefonte, PA, USA) were used. Contaminated corn and unreconstituted milk powder samples (50 g) were analyzed in triplicate. The corn samples were mixed with a solution of methanol–water (8:2, 100 mL) and was processed using tube solid phase extraction (SPE). The SPE was washed with tetrahydrofluoran (THF, 20% 0.5 mL), hexane (2.0 mL), and THF (25%, 3.0 mL). Methylene chloride (2 mL) was used as an elution solution with THF (99:1). The eluate obtained from the SPE was evaporated, and the residue was suspended in methanol (100 µL); thereafter, acetic acid was added (0.5%, 100 µL). AFs were derivatized with trifluoroacetic acid and injected into a chromatographic system (binary pump and fluorescence detector; LC–18 column, 25 cm × 4.6 mm, packing 5.0 μm; LC-18 guard column, 2.0 cm × 4.6 mm, same packing). HPLC was performed under the following conditions: 360 nm excitation and 440 nm emission for fluorescence detection; acetonitrile–methanol–water (1:1:2, *v*/*v*/*v*) for mobile phase; 1.0 mL/min of flow rate; 20 µL of injected sample on methanol–acetic acid 0.5% (1:1); and 25 °C ± 2 °C of temperature. The limit of detection was estimated for AFs was 2.0 ng/g.

In addition, the milk and feed ingested by newborn calves were analyzed using the ELISA method to verify the absence of AFM1 and other mycotoxins (detection limit < 0.08 µg/L). Corn and concentrate feed samples were also analyzed for ochratoxin (OTA), fumonisins (FBs), zearalenone (ZEA), deoxynivalenol (DON), and aflatoxin M1 (AFM1) in milk using competitive ELISA kits (Absorbance Microplate Reader, BioTek Instruments, Winooski, VT, USA; Diagnostic kit Ridascreen Fast, R-Biopharm AG, Darmstadt, Germany: OTA, FBs, ZEA, DON, and AFM1).

### 2.3. Biochemical and Pathological Analyses

Biochemical studies in plasma included total proteins (TP), albumin, (ALB), reduced glutathione (GSH), total and direct bilirubin (TB and DB), prothrombin time (PT), alkaline phosphatase (ALP), gamma-glutamyltransferase (GGT), and alanine and aspartate aminotransferases (ALT and AST) measured at 1, 7, 15, and 30 dpe. In tissues, levels of GGT, GSH, and glutathione S-transferases (GST) were measured. The GGT activity was measured in urine. Diagnostic kits for ALB, bilirubin, ALP, ALT, AST, and GGT (BioSystems, Barcelona, Spain) and PT (BioMérieux, Durham, NC, USA) were used; GSH concentrations were measured by the fluorometric method (spectrofluorometer Perkin Elmer LS50B, Norfolk, CA, USA) [18], and GST was measured by spectrophotometric analysis (spectrophotometer Varian DMS-80, Varian Aas., Inc., Victoria, Australia) [19].

All calves were euthanized, dividing 5 calves from both the NE-G and the E-G at each of days 7, 15, and 30 post exposure, in accordance with the country’s official regulations [20]. The liver and kidney were collected, weighted, and observed to detect macroscopic alterations. Liver and kidney lobe samples for histopathological analysis were preserved in neutral formalin (10%) according to standard procedures. Masson’s trichrome, periodic acid–Schiff, and hematoxylin and eosin stains were used for histological analysis.

### 2.4. Statistical Analysis

The data obtained from continuous variables (DM intake, BW gain, etc.) were analyzed using the independent samples *t*-test between the E-G and NE-G groups under repeated measurements over time after exposure (comparison of the mean values of NE-G vs. E-G at 1, 7, 15, and 30 dpe). Significant differences (*p* < 0.05) were identified using a 95% CI for binomial proportions in each post exposure date. Categorical data for macroscopic and microscopic alterations were analyzed with an uncorrected chi-squared test (χ^2^) at a significance level of *p* < 0.05. Histological analysis was performed independently (blinded) by two external pathologists, and the tissues were explored looking for a homogeneous distribution of lesions in the hepatic lobes or renal regions, so each sample (in triplicate) was considered a single unit, and the results were reported from the corresponding sample size.

## 3. Results

### 3.1. Animal Performance

None of the exposed animals exhibited alterations before exposure to mycotoxins, so their development was normal according to their age; the NE-G animals remained healthy for 30 days after exposure. However, at 3 dpe, E-G animals showed depression, immobility, rough hair, nasal discharge, and decreased body condition; they also exhibited diminished performance in DMI, BWG, and GR. These differences were observed from the first week (Table 1) and worsened by the end of the observation period (30 days). NE-G showed up to 2.3 times higher DMI (*p* < 0.01) than E-G (10.7 ± 0.0 vs. 4.5 ± 0.08 kg/week, respectively). Similarly, a decrease in BWG (*p* < 0.01) was observed in E-G, reaching 1.5 times less than NE-G (73 ± 0.0 vs. 48.2 ± 4.8 kg/week, respectively). Because of the decreases in DMI and BWG, GR showed a significant decrease (*p* < 0.05; 0.15 ± 0.0 vs. 0.09 ± 0.01 DMI/BWG, respectively).

### 3.2. Biochemistry

Single exposure to AF-contaminated feed was associated with characteristic and significant biochemical alterations. Some variables (TP, DB, and TB) showed constant changes throughout the study period. However, enzyme activity, ALB, and PT showed a biphasic curve, with greater changes in the initial phase of the study (Table 2) and a gradual return to normal values before 30 days. In this sense, E-G showed a progressive decrease in TP compared with NE-G (*p* < 0.01), with this trend reduced by 56.0 ± 8.2% during the study period. Plasma ALB decreased during the first half of the study (*p* < 0.05; 38.6 ± 4.8%) in E-G compared with NE-G. The bilirubin levels were increased during the entire study period. DB and TB concentrations increased by 8.2–16.6- and 1.9–17.9-fold, respectively, in E-G compared with the nonexposed group (*p* < 0.01).

Moreover, in the exposed calf group, the plasma-specific activities of ALP, AST, and ALT enzymes showed significant increases (*p* < 0.05) as compared with NE-G. The increase in AST activity was observed late (7–30 dpe), while changes in ALT and ALP occurred immediately in the first half of the study. The AST, ALT, and ALP activities in E-G were relative increased in comparison with NE-G (2.6–4.4, 2.1–4.7, and 1.7–2.3 times, respectively). Plasma activity of the GGT enzyme in E-G showed a greater increase in the initial phase of the study (1 and 7 dpe; 1.8–2.0 times; *p* < 0.05); later, the values resembled those of NE-G. The coagulation process was also altered, and the PT was increased 1.2–1.3 times in the first week (*p* < 0.05), although the increase was not significant thereafter.

### 3.3. Tissue Biochemistry

The biochemical functions of the liver and renal tissues involved in the process of elimination of mycotoxins showed noticeable changes in the E-G animals (Table 2). Tissue GSH levels increased in both tissues in response to contaminated feed exposure; this compound had the highest concentration at 15 dpe and declined to normal levels by the end of the study (30 dpe). The return to normal GSH values coincided with an increase in plasma GST activity, which is involved in the process of the elimination of AFs. Urinary GGT had significant increases (*p* < 0.01) throughout the study (E-G/NE-G: 205/77.8 and 51.1/10.0 U/L at 7 and 30 dpe).

### 3.4. Morphological Findings

**Figure 1:**
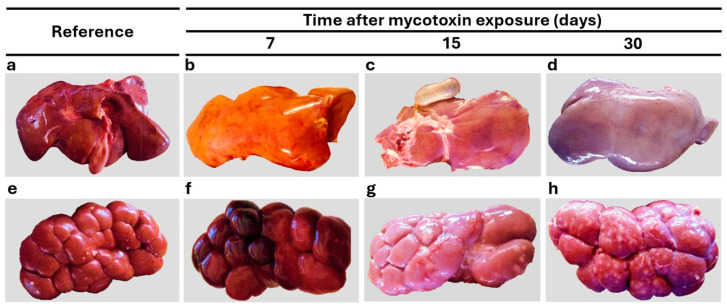
Characteristic morphological alterations in the liver and kidney. (**a**) Liver without gross lesions and with characteristic coloration. Nonexposed group. (**b**) Liver with yellowish discoloration and friable consistency. (**c**) Liver with multifocal hemorrhages and friable consistency. (**d**) Liver with pale discoloration and hemorrhagic foci. (**e**) Kidney without gross lesions and with characteristic appearance. (**f**) Hemorrhagic kidney. (**g**) Kidney with mottled appearance and firm consistency. (**h**) Multiple white-gray foci in the kidney.

**Figure 2:**
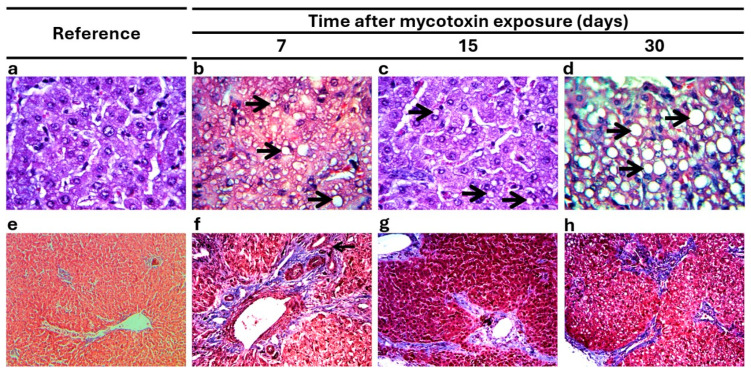
Typical microscopic alterations in liver. (**a**) Liver tissue without microscopic changes (nonexposed group, H & E stain, 40×). (**b**) Liver showing macrovesicular steatosis (arrowhead) with generalized distribution (H & E, 40×). (**c**) Liver showing microvesicular steatosis (arrowhead) with a minor distribution (H & E, 40×). (**d**) Liver showing diffuse macrovesicular steatosis (arrowhead) (H & E, 40×). (**e**) Liver periportal tissues without microscopic changes (Masson’s trichrome stain, 10×). (**f**) Hepatic periportal fibrosis (Masson’s trichrome, 10×). (**g**) Liver fibrosis with interlobular septal thickening and wide distribution. (**h**) Hepatic periportal fibrosis (Masson’s trichrome, 10×).

**Figure 3:**
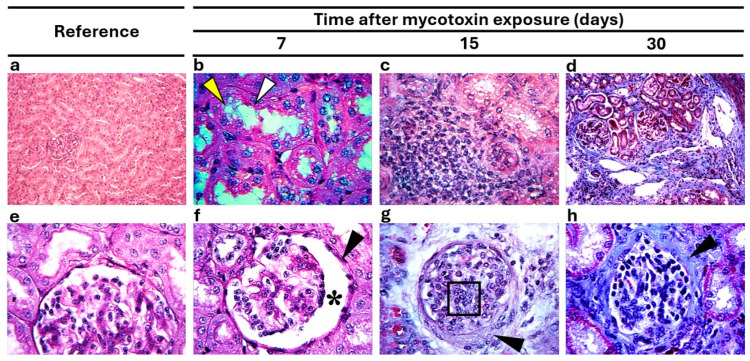
Typical microscopic alterations in kidneys. (**a**) Renal tissue without microscopic changes in tubules and glomeruli (H & E, 10×). (**b**) Kidney with loss of the brush border (arrowheads) in proximal tubules (PAS, 40×). (**c**) Foci of lymphocytic infiltration in the tubular area (PAS, 40×). (**d**) Fibrosis in renal parenchyma and loss of normal architecture (Masson’s trichrome, 10×). (**e**) Renal tissue without microscopic changes in renal tubules and glomeruli (PAS, 40×). (**f**) Kidney with glomerular atrophy, increased Bowman’s space (asterisk), and thickening (arrowhead) of the capsules of parietal cells (Masson’s trichrome, 10×). (**g**) Kidney showing (arrowhead) sclerotic glomerulonephritis and lymphocytic infiltration (black square) (periodic acid–Schiff, 40×). (**h**) Kidney with thickening (arrowhead) in Bowman’s capsule (Masson’s trichrome, 40×).

In accordance with the biochemical changes, exposure to mycotoxin-contaminated feed in calves was associated with several morphological and microscopical alterations, especially in the liver and kidney. However, the NE-G did not exhibit any liver or kidney changes (Figure 1a, Figure 1e, Figure 2a, Figure 2e, Figure 3a and Figure 3e, respectively). Macroscopic alterations were observed during all three observation periods (7, 15, and 30 dpe) (Table 3). No significant differences in absolute and relative liver or kidney weights were observed between E-G and NE-G. Macroscopic alterations detected in the liver and kidney showed a time-dependent pattern (Figure 1); nevertheless, in E-G, the liver showed yellowish color with greasy aspect, hemorrhagic biliary bladder and abnormal changes in bile color (7 dpe) (Figure 1b), and friable consistency with multifocal hemorrhages at 15 dpe (Figure 1c). Later, the color of the liver was not observed with a greasy aspect but with a pale discoloration at 15 and 30 dpe (Figure 1c and Figure 1d, respectively). In E-G, kidneys were hemorrhagic and had friable consistency at 7 dpe (Figure 1f). However, at 15 and 30 dpe, hemorrhages were less frequent, and the kidneys appeared pale, with firm consistency and mottled appearance (Figure 1g and Figure 1h, respectively). The statistical analysis of macroscopic lesion frequency in the liver and kidney could not be performed because several of the findings had fewer than five lesions.

Microscopic analysis of the liver and kidneys provided additional evidence of lesions in cattle exposed to AFs (Table 4). The most frequent alterations observed in the liver and kidneys included lymphocyte infiltration, accumulation of eosinophilic material, fibrosis, and cell degeneration. The frequency and severity of microscopic alterations in the liver and kidneys increased during the observation period in E-G (three weeks, χ^2^ < 0.01). There were no significant microscopic changes in NE-G (Figure 2a,e and Figure 3a,e). Fatty liver was observed in E-G, related to the macroscopic yellowish appearance. Hepatic steatosis was observed as the accumulation of lipids in the form of globules surrounding the cytoplasm of hepatocytes, altering the contour of the cell. Steatosis followed this pattern during the three observation periods: microvesicular with a generalized distribution (7 dpe; Figure 2b), microvesicular with a minor distribution (15 dpe; Figure 2c), and macrovesicular with a generalized distribution (30 dpe; Figure 2d). Fibrosis was also present, altering the cellular organizations characteristic of hepatic tissue and was coincident with firmer consistency of this organ. Histologically, fibrosis was observed as the deposition of excess collagen fibers with a variable distribution according to the observation period (Figure 2f–h): fibrosis was perivascular at 7 dpe (Figure 2f), while at 15 (Figure 2g) and 30 dpe (Figure 2h), it was widely distributed, with interlobular septal thickening. This finding coincided with the firmer consistency of this organ observed at 15 and 30 dpe. There were other lesions with a time-dependent pattern, such as perivascular and interstitial lymphocyte infiltrations, with increased extension at later periods. The biliary ducts showed inflammation and obstruction, along with increased numbers of ducts in the right, left, and quadrate lobes of the liver (7, 15, and 30 dpe).

In the kidneys, alterations were observed in tubules and glomeruli in the E-G (Figure 3). In the tubular area, loss of the brush border in proximal tubules (Figure 3b), foci of lymphocytic infiltration (Figure 3c), and fibrosis with a presentation pattern like that of hepatic fibrosis (Figure 3d) were observed during the three observation periods (7, 15, and 30 dpe). Glomerular atrophy was also observed in the kidneys, with increased Bowman’s space (Figure 3f); additionally, there was thickening of parietal cells in Bowman’s capsule throughout the study period. There was also increased mesangial matrix and collagen deposits in glomeruli and increased capillary membrane was also present (Figure 3g). Renal architecture was altered by the presence of fibrosis, and it has the same time pattern observed in liver at 7, 15 y 30 dpe (Figure 3h).

## 4. Discussion

This study analyzed the effects a single exposure of Holstein male calves to feed contaminated with a high concentration of AFs. This exposure led to the development of a characteristic time pattern of clinical manifestations, biochemical changes, and pathological lesions consistent with the appearance of acute aflatoxicosis syndrome. Calves exposed to mycotoxins in feed showed severe alterations in productive performance in comparison with unexposed animals. The exposed calves showed transient or persistent biochemical changes. In addition, these performance and biochemical changes were related to macroscopic and microscopic morphology alterations in the liver and kidneys. Several of our results agree with previous works about the acute aflatoxicosis syndrome in bovine [21,22,23]. However, it is our knowledge that many of these time-dependent alterations in biochemistry and histology during single AF exposure are published here for the first time. These findings are relevant to making a proper diagnosis of acute aflatoxicosis in cattle, as well as to designing relevant measures to protect public health and the dairy industry.

Zearalenone in feed and AFM1 in milk were estimated at relatively low amounts (16.3 ± 8.2 μg/kg and 8.0 ± 2.5 ng/L), while no appreciable concentrations of DON, FBs, or OTA were detected. Single exposure to AF-contaminated feed in dairy calves produced several significant time-dependent changes in animal performance and plasma biochemistry; necropsy showed characteristic lesions and multiple histological alterations in the liver and kidneys. In addition, the biochemistry of liver and kidney tissues showed AF-induced alterations. In this study, poor performance was observed in E-G animals, especially in the GR, BWG, and DMI indicators. It has been suggested that the reduction in performance produced by aflatoxicosis is a multifactorial effect involving both damage to body tissues and alterations in digestive metabolism. These alterations include malabsorption of macronutrients; low utilization of proteins, carbohydrates, and lipids; reduced bile acid concentration; and decreased enzymatic activity of amylase, trypsin, and lipase. Additionally, decreased glycogenesis and accelerated glycogenolysis contribute to decreased growth rate and body weight [24,25,26,27]. This background suggests that the decrease in growth observed in this study was caused by both the direct toxic effect of AFs and a decrease in systemic metabolism, resulting in impaired digestive functionality and low nutrient utilization.

In the initial period of the study, we observed that the increase in PT in E-G animals coincided with a coagulopathy process that may have been due to impaired blood coagulation and decreased protein synthesis in the liver [21]. These changes coincided with diminished PT and ALB levels, along with increased DB and TB levels. All these changes may have been due to impaired globulin synthesis, hepatic lesions, and blockage of biliary circulation, leading to an influx of bilirubin conjugates into the blood vessels [12]. Subsequently (15 and 30 dpe), a decrease in severity of morphological alterations coincided with increases in TP and ALB, as well as with a tissue decrease in GSH and GST, suggesting the existence of a partial recovery process of liver function [5]. It has been proposed that GSH plays a regulatory role in AF toxicity through the formation of AFBO–GSH conjugates and their subsequent elimination [22]; thus, GSH concentration in the plasma, liver, and kidney is related to the intracellular utilization of GSH in the AF target organs of intoxicated animals. The activity of some enzymes in urine and plasma reflects the damage produced in the whole organism; however, quantification of enzyme activity directly in organ tissue could provide more precise information on their responsiveness to the presence of toxic agents such as AFs. In our study, the increase in GSH in the liver and kidneys observed between 15 and 30 dpe suggests a compensatory effect of the organs involved in the synthesis of this antioxidant and in the PA detoxification pathway. Furthermore, in this study, a decrease in renal GST activity was observed at the end of the monthly observation period. GST has been shown to be a very important isoenzyme for detoxification processes, as it catalyzes the conjugation of GSH with AFs and other electrophilic molecules derived from toxic and carcinogenic compounds [5], in addition to the fact that its enzymatic activity is stimulated by the presence of elevated levels of AFs and intracellular GSH [28,29]. In our study, AFs did not induce a decrease per gram of tissue in hepatic or renal GST catalytic activity in the E-G group, suggesting that the lack of specific enzyme activity could have been due to the decrease in total protein synthesis in both organs caused by the toxic effect of AFs. GGT activity was quantified in the liver and kidney. Calves in the E-G group showed an increase in GGT activity from 15 dpe onwards, with the activity being much higher in the kidneys. GGT functions have been associated with GSH metabolism by mobilizing the gamma-glutamyl group and allowing reuse of the constituent amino acids for GSH synthesis [30]. Thus, increased GGT enzyme activity in serum and urine indicates liver and kidney damage, respectively [13]. This increase in GGT enzyme activity coincided with the liver and kidney lesions observed in the E-G calves in this study, such as glomerulonephritis and loss of the brush border of proximal renal tubules. Additionally, increases in serum GGT, ALP, AST, and ALT enzyme activities were observed from the onset of exposure to the AFs. These enzymes are intracellular or localized in the plasma membrane of hepatocytes or in the brush border cells of renal proximal tubules. Therefore, their activity in serum and urine is associated with the extent of AF-induced liver damage, including inflammation, necrosis, proliferation, and thickening of bile ducts with cholestasis [12,22].

In this study, calves in E-G were exposed to a single dose of AFs, and no significant differences in relative liver and kidney weights were observed. Studies with prolonged exposure to these mycotoxins have reported the development of liver hyperplasia, bile duct proliferation, fibrosis, and hypertrophy of the smooth endoplasmic reticulum of hepatocytes, all of which result in increased organ size [13,31]. Additionally, in our study, at 7 dpe, fatty degeneration and friable consistency were observed in the liver of calves in E-G. Microscopically, hepatic fatty degeneration was observed as microvacuolar steatosis at 7 dpe and as macrovacuolar steatosis at 30 dpe. The fatty appearance of the liver produced by AFs has been attributed to altered lipid metabolism, as AFs interfere with the function of genes involved in the synthesis of fatty-acid-metabolizing enzymes in the liver, resulting in decreased lipid enzyme activity, fatty acid transport, and increased fatty acid concentration in hepatocytes [32,33].

Similarly, single exposure to AFs produced fibrosis in the portal tract and hepatic parenchyma of calves during a postexposure period of 30 days. It has been proposed that fibrosis occurs in response to significant cell destruction associated with inflammatory processes in tissues that do not show adequate regeneration, thus resulting in replacement of the damaged tissue with collagen fibers [33,34,35]. Thus, the frequent presence of hepatic and renal fibrosis observed in E-G suggests that the cellular degeneration caused by AFs induced a chronic inflammatory process and collagen production in both organs. The severity of fibrosis increased over time, along with the presence of foci of lymphocytic infiltration that persisted throughout the experimental phase. Other relevant renal alterations observed in E-G included glomerulonephritis, inflammation of Bowman’s capsule, and glomerular atrophy. The susceptibility of the kidneys to various xenobiotics such as AFs, which are distributed or eliminated through the bloodstream, has been highlighted because of the large amount of blood these organs receive and process. As a result, nephrons are exposed to high concentrations of toxicants and their metabolites producing various lesions such as thickening of the glomerular basement membrane, abnormal development of glomerular epithelial cells, and degenerative changes in the tubular cells [23,36,37].

Glomerulopathies have been reported in experimental AF intoxications, characterized by corpuscular changes such as increased urinary space due to compensatory hypertrophy [38,39,40]. Additionally, the thickening of the glomerular basement membrane decreases the glomerular filtration rate and the rate of toxin clearance, resulting in increased exposure to AFs [36]. These changes could serve as valuable indicators of the alterations induced by acute aflatoxin poisoning in bovines. Proper diagnosis of aflatoxicosis is crucial in the cattle industry to establish measures that mitigate its negative impact on the health and productive capacity of cattle [14,15,41].

In summary, this study describes the toxic effects caused in calves by a single exposure to aflatoxin-contaminated feed. Compared with unexposed calves, exposed calves showed a sequence of time-dependent changes in performance (body weight gain, dry matter intake, feed conversion ratio), blood biochemistry (protein, bilirubin, reduced glutathione, etc.), serum and tissue enzyme activity (gamma-glutamyltransferase, glutathione-S-transferase, aspartate aminotransferase, etc.), and macro- (hemorrhage, coloration, consistency) and microscopic morphology (steatosis, fibrosis, glomerulonephritis, etc.). These results suggest that the identification of alterations and time-dependent sequences in animal performance and in biochemical and morphological characteristics can be useful to integrate an adequate diagnosis of bovine aflatoxicosis.

## Figures and Tables

**Table 1:** Time-dependent changes in performance (mean ± SEM) of male Holstein calves exposed to mycotoxin in concentrated feedstuffs.

Performance ^a^	Reference ^b^	Time After Mycotoxin Exposure (Days)			
		1	7	15	30
**DMI** (kg/d)	7.0–7.6	4.9 ± 0.42 **	5.71 ± 1.14 *	5.3 ± 1.17 *	4.54 ± 0.08 **
**Milk** (kg/d)	6.0–6.1	2.9 ± 0.1 4 **	5.8 ± 0.3	4.6 ± 0.6 **	4.2 ± 0.3 **
**BW** (kg)	59.5–62.3	50 ± 2.17 **	58.3 ± 7.8	59.4 ± 11.6	48.2 ± 4.8 **
**CI** (DMI/BW)	0.91–1.37	0.73 ± 0.23 *	1.36 ± 1.14	4.82 ± 1.17 **	−4.54 ± 0.08 **

^a^ DMI: daily dry matter intake; Milk: daily milk intake; BW: body weight; CI: conversion index. ^b^ Range of values observed in the nonexposed group. *, **: *p* < 0.05 and *p* < 0.01 significant differences between reference and day means (±SEM).

**Table 2:** Time-dependent changes in plasma and tissues biochemical values (mean ± SEM) of male Holstein calves exposed to mycotoxin in concentrated feedstuffs.

Biochemistry ^a^	Reference ^b^	Time After Mycotoxin Exposure (Days)			
		1	7	15	30
***Plasma***					
**ALB** (g/dL)	2.7–2.7	2.0 ± 0.28 *	1.34 ± 0.3 *	1.6 ± 0.2 *	1.53 ± 0.04 *
**TP** (g/dL)	15.6–16.7	7.3 ± 0.49 **	6.1 ± 0.1 **	6.9 ± 0.8 **	11.5 ± 1.20 **
**DB** (mg/dL)	0.31–0.37	1.30 ± 0.12 *	5.3 ± 0.0 **	6.4 ± 0.5 **	9.4 ± 0.79 **
**TB** (mg/dL)	1.75–1.82	2.9 ± 0.14 *	35.1 ± 0.3 **	36.6 ± 0.3 **	44.2 ± 1.65 **
**PT** (s)	19.0–19.3	20.9 ± 0.73	19.7 ± 0.8	20.6 ± 1.0	22.5 ± 1.00
**ALT** (EA, U/L)	15.0–23.8	49.7 ± 6.2 *	58.0 ± 12.4 *	41.6 ± 9.4 *	41.9 ± 11.9 *
**AST** (EA, U/L)	56.5–64.5	49.7 ± 21.2	286 ± 61.0 **	228 ± 34.3 **	218 ± 0.00 **
**ALP** (EA, U/L)	71.9–80.0	116 ± 13.5 *	186 ± 14.4 **	144 ± 21.2 **	104 ± 4.2 *
**GGT** (EA, U/L)	118–142	157 ± 22.7 *	304 ± 58.1 **	185 ± 31.4 *	150 ± 6.0 *
***Liver***					
**GSH** (μmol/g)	0.66 ± 0.11	0.87 ± 0.20	0.98 ± 0.41 *	1.26 ± 0.15 **	1.26 ± 0.15 **
**GST** (μmol CDNB/min)	45.6 ± 0.11	43.0 ± 0.15	42.8 ± 2.68	45.5 ± 2.3	36.7 ± 0.15 *
**GGT** (AE, U/g)	49.2 ± 1.25	35.0 ± 2.0 *	50.1 ± 10.4	363 ± 178 **	230 ± 23.5 **
***Kidney***					
**GSH** (μmol/g)	0.76 ± 0.01	0.78 ± 0.03	1.23 ± 0.37 *	1.26 ± 0.05 **	0.76 ± 0.01
**GST** (μmol CDNB/min)	44.4 ± 0.53	45.0 ± 0.90	44.8 ± 2.2	38.2 ± 3.5	29.0 ± 0.75 *
**GGT** (AE, U/g)	95.8 ± 1.50	58.0 ± 5.3 *	250 ± 104 **	597 ± 124 **	776 ± 73.5 **

**^a^** TP: serum total protein; ALB: serum albumin; DB: direct bilirubin; TB: total bilirubin; AST: aspartate aminotransferase; ALT: alanine aminotransferase; ALP: alkaline phosphatase; GGT: gamma glutamyltransferase; PT: prothrombin time; EA: specific enzyme activity; GSH: reduced glutathione; GST: glutathione S-transferase. ^b^ Range of values observed in the nonexposed group. *, **: *p* < 0.05 and *p* < 0.01 significant differences between reference and day means (±SEM).

**Table 3:** Gross alterations detected in male Holstein calves exposed to mycotoxin in concentrate feed (exposed group) compared with the nonexposed group.

Morphological Findings	Nonexposed Group					Exposed Group				
	Days Postexposure			(No.)	(%)	Days Postexposure			(No.)	(%)
	7	15	30			7	15	30		
***Liver***										
Pallor	0	0	0	0/15	0.0	5	5	5	15/15	100
Hepatomegaly	1	0	0	1/15	6.7	0	5	2	7/15	46.7
Hemorrhages	1	0	0	1/15	6.7	5	5	5	15/15	100
Friable consistency	0	0	0	0/15	0.0	5	5	5	15/15	100
***Kidney***										
Pallor	0	0	0	0/15	0.0	0	1	3	4/15	26.7
Greyish-white foci	0	0	1	1/15	6.7	5	4	0	9/15	60.0
Hemorrhages	1	0	0	1/15	6.7	5	4	0	9/15	60.0
Friable consistency	0	0	0	0/15	0.0	5	5	0	10/15	66.7

**Table 4:** Microscopic alterations detected in tissue samples of the liver and kidney of male Holstein calves exposed to mycotoxins in concentrate feed compared with the nonexposed group.

Morphological Findings	Nonexposed Group ^a^		Exposed Group ^a^		χ^2^	*p*- Value	OR
	(No.)	(%)	(No.)	(%)			
***Liver***							
Bile duct obstruction	1	2.2	43	95.6	78.4	<0.01	946
Cell degeneration	3	6.7	43	95.6	71.1	<0.01	301
Cholestasis	0	0	30	66.7	45.0	<0.01	*
Eosinophilic material	1	2.2	23	85.2	27.5	<0.01	46.0
Fatty degeneration	0	0	30	66.7	45.0	<0.01	*
Fibrosis	0	0	39	86.7	68.8	<0.01	*
Lymphocytic infiltration	1	2.2	24	88.9	29.3	<0.01	50.3
***Kidney***							
Brush border edge loss	0	0	45	100	90.0	<0.01	*
Cell degeneration	1	2.2	40	88.9	68.1	<0.01	352
Eosinophilic material	3	6.7	42	93.3	67.6	<0.01	196
Fibrosis	0	0	35	77.8	57.3	<0.01	*
Foci of bleeding	2	4.4	40	88.9	64.5	<0.01	172
Glomerular atrophy	0	0	25	55.6	34.6	<0.01	*
Glomerulonephritis	0	0	23	51.1	30.9	<0.01	*
Lymphocytic infiltration	1	3.7	27	60	35.0	<0.01	66.0
Mesangial proliferation	0	0	43	95.6	82.3	<0.01	*
Pigmentation	0	0	39	86.7	68.8	<0.01	*
Thickening of blood vessels	1	2.2	42	93.3	74.9	<0.01	616
Thickening of Bowman’s capsules	0	0	43	95.6	82.3	<0.01	*
Vacuolization	0	0	20	44.4	25.7	<0.01	*

^a^ Each sample was made by two subsamples in three organic regions (*n* = 45 by group). * Odds ratio with positive infinite value by division of the value in the EG over zero in the NE-G.

## Data Availability

The original contributions presented in this study are included in the article. Further inquiries can be directed to the corresponding author.
